# ehealth technology in cardiac exercise therapeutics for pediatric patients with congenital and acquired heart conditions: a summary of evidence and future directions

**DOI:** 10.3389/fcvm.2023.1155861

**Published:** 2023-06-02

**Authors:** David A. White, Aimee M. Layton, Tracy Curran, Naomi Gauthier, William B. Orr, Kendra Ward, Meg Vernon, Matthew N. Martinez, Malloree C. Rice, Katherine Hansen, Megan Prusi, Jesse E. Hansen

**Affiliations:** ^1^ Ward Family Heart Center, Children’s Mercy Kansas City, Kansas City, MO, United States; ^2^ School of Medicine, University of Missouri Kansas City, Kansas City, MO, United States; ^3^ Division of Pediatric Cardiology, Department of Pediatrics, Columbia University Irving Medical Center, New York, NY, United States; ^4^ Department of Cardiology, Boston Children’s Hospital, Boston, MA, United States; ^5^ Division of Pediatric Cardiology, Department of Pediatrics, Washington University School of Medicine, St. Louis, MO, United States; ^6^ Division of Cardiology, Department of Pediatrics, Ann & Robert H. Lurie Children’s Hospital of Chicago, Chicago, IL, United States; ^7^ Division of Cardiology, Department of Pediatrics, Seattle Children’s Hospital, Seattle, WA, United States; ^8^ Division of Pediatric Cardiology, Department of Pediatrics, Hassenfeld Children’s Hospital at NYU Langone, New York, NY, United States; ^9^ Division of Pediatric Cardiology, Heart Institute, Cincinnati Children’s Hospital Medical Center, Cincinnati, OH, United States; ^10^ Division of Pediatric Cardiology, Department of Pediatrics, C.S. Mott Children’s Hospital, Ann Arbor, MI, United States

**Keywords:** pediatric cardiology, exercise prescription, cardiac rehabilitation, technology, wearable devices, telehealth

## Abstract

Many children and adolescents with congenital and acquired heart disease (CHD) are physically inactive and participate in an insufficient amount of moderate-to-vigorous intensity exercise. Although physical activity (PA) and exercise interventions are effective at improving short- and long-term physiological and psychosocial outcomes in youth with CHD, several barriers including resource limitations, financial costs, and knowledge inhibit widespread implementation and dissemination of these beneficial programs. New and developing eHealth, mHealth, and remote monitoring technologies offer a potentially transformative and cost-effective solution to increase access to PA and exercise programs for youth with CHD, yet little has been written on this topic. In this review, a cardiac exercise therapeutics (CET) model is presented as a systematic approach to PA and exercise, with assessment and testing guiding three sequential PA and exercise intervention approaches of progressive intensity and resource requirements: (1) PA and exercise promotion within a clinical setting; (2) unsupervised exercise prescription; and (3) medically supervised fitness training intervention (i.e., cardiac rehabilitation). Using the CET model, the goal of this review is to summarize the current evidence describing the application of novel technologies within CET in populations of children and adolescents with CHD and introduce potential future applications of these technologies with an emphasis on improving equity and access to patients in low-resource settings and underserved communities.

## 1. Introduction

Providers of patients with congenital and acquired heart conditions (CHD) have an increasingly broad range of approaches available to facilitate engagement in habitual physical activity (PA) and exercise to improve cardiovascular health outcomes. Historically, it was common for patients to be restricted from participating in recreational PA and exercise by medical providers due to anecdotal concerns about cardiovascular risk (1, 2). Avoidance of PA and exercise was often been amplified by patients self-restricting their PA, or restrictions imposed on patients by their parents or other caretakers such as teachers and coaches, due to fear and uncertainty about the child's heart disease (1, 3–6). It should be recognized that some patients may have diagnoses-specific indications where modifications or limitations to certain types of exercise is warranted (4, 7). However, evidence over the past 25 years has led to increased recognition amongst providers that an individualized PA and exercise intervention for patients with CHD provides cardiovascular, social, academic, and quality of life (QoL) benefits that outweigh historical theoretical and anecdotal risks (8–11).

Longitudinal studies suggest the greatest reductions in CHD related morbidities and mortality are derived from improvements in cardiorespiratory fitness (12–17). While the terms *exercise* and PA are often used as surrogates of fitness, exercise and PA are only contributors to fitness and have different meanings. PA is defined as *any* bodily movement that requires energy expenditure, where the intensity of the movement is not a moderating factor (4, 18). Exercise, a subcategory of PA, is structured, repetitive, purposeful, and typically of a moderate-to-vigorous intensity, with the intention of enhancing fitness and health (4, 18, 19). Exercise and PA can be influenced by an individual's knowledge, self-efficacy toward PA and exercise, and motivation. Fitness is a representation of physiologic reserve, characterized by an individual's ability to perform PA and exercise, and is mediated by hemodynamic and genetic factors (1, 10, 20).

The multi-institutional Technology Evaluation and Usage in Cardiac and Fitness Rehabilitation (TECH) working group of the Global Coalition for Fitness and CHD (GloCo) set out to review current needs and objectives for technology for our field and our patients. GloCo was developed to unite as a global coalition of healthcare providers dedicated to evaluating and optimizing overall fitness of hearts and minds of patients living with congenital and pediatric acquired heart conditions. The aim of GloCo is to outline fitness assessments, interventions, and outcomes, with an emphasis on collaboration and research, raising awareness and empowering patients and families, and identifying the role and use of technology for PA promotion and exercise intervention (see the acknowledgements section for more information about GloCo).

The GloCo TECH working group defined “cardiac exercise therapeutics” (CET) as a broad and novel term representing a range of PA and exercise assessments and interventions that may be considered for patients with CHD to improve cardiorespiratory fitness. The CET spectrum includes PA and fitness assessments, general promotion of PA, targeted exercise prescriptions, to highly structured and supervised fitness training interventions (i.e., formal cardiac rehabilitation). New and emerging eHealth (electronic delivery of health services) and mHealth (mobile health) technologies have the potential to enhance access, reduce barriers to implementation, and improve the effectiveness of CET.

In this review, we aim to use the CET model to discuss eHealth technologies relevant to enhancing assessment/testing and intervention approaches. eHealth is broadly defined as the application of technology and the internet for: (1) the delivery of healthcare; and/or (2) patient monitoring (21). We begin by introducing the CET model. The subsequent sections outline each sub-component of the CET model, including a discussion on the applications of eHealth, mHealth, and other technologies in the CHD population as well as applications to other relevant patient populations. We will conclude by discussing issues related to equity and accessibility as it relates to technology in CET and propose future research directions related to application of technology in CET.

## 2. Systematic approach to cardiac exercise therapeutics (CET)

**Figure 1:**
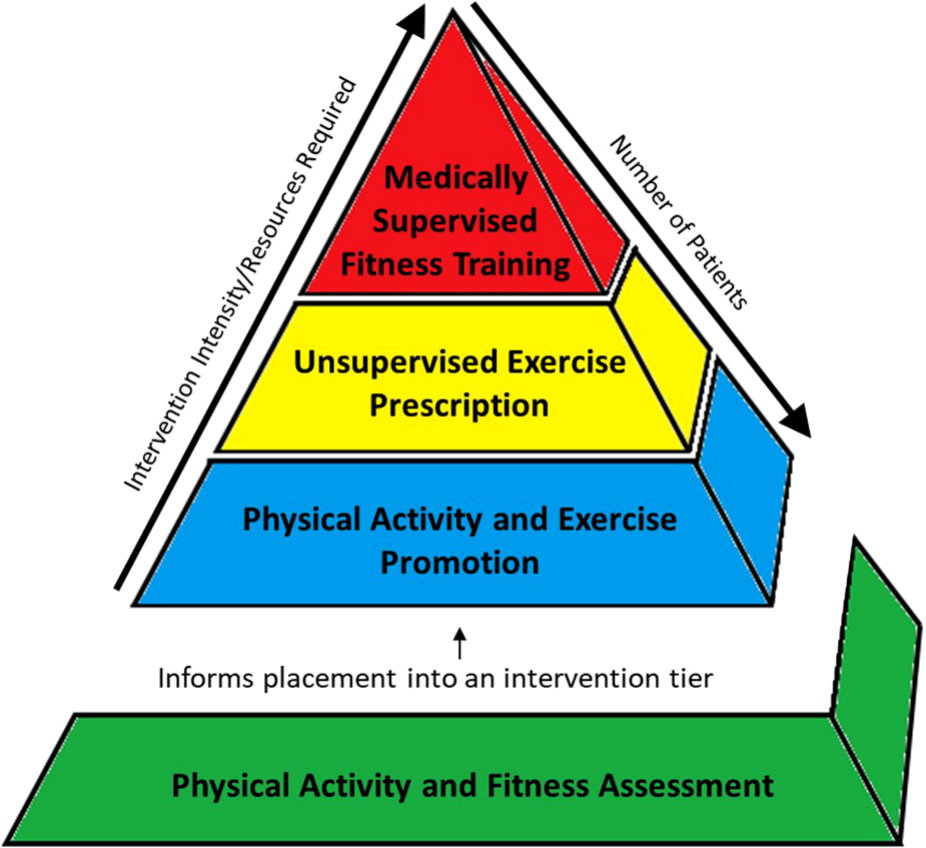
Cardiac exercise therapeutics (CET) model.

The CET model includes assessment and testing to guide a patient's placement in one of three intervention approaches: (1) general PA promotion strategies; (2) exercise prescription; and (3) structured and supervised fitness training interventions. Figure 1 is a representation of the CET model in which the bottom level of the pyramid represents PA and exercise assessments that inform appropriate intervention selection for each patient. In this model, intervention tiers at the bottom of the pyramid (i.e., PA and exercise promotion) are broadly applicable, require lower resource utilization, but have the greatest potential to be disseminated amongst large numbers of patients; whereas the top of the pyramid (i.e., fitness training interventions) are more focused and individualized, require high resource utilization, and may be utilized in a small number of patients with the greatest needs.

**CET Model:**

- All CHD Patients
  • **Physical Activity and Fitness Assessment:** PA and Fitness Assessments utilize objective monitoring of PA participation and fitness testing to identify if the patient is meeting PA recommendations and/or evaluate degree of fitness impairment. The result of this step provides data to guide the initial assignment to one of the three intervention tiers.
- Intervention Tiers
  • **Physical Activity Promotion:** The most prevalent need is for PA promotion, which represents educating and encouraging the patient and family to participate in habitual and lifestyle PA in accordance with public health guidelines and CHD-specific recommendations.
  • **Unsupervised Exercise Prescription:** Exercise prescription refers to the formulation of a provider or exercise physiologist derived exercise prescription which outlines the frequency, intensity, duration, and modality of a self-directed and unsupervised exercise routine or program that is performed by the patient in their homes or communities.
  • **Medically Supervised Fitness Training (MSFT):** The top of the pyramid is structured and supervised fitness training, the classic example being cardiac rehabilitation (CR). This level may only be utilized by a select group of patients with the greatest needs, requiring the most supervision and expertise and is only available in certain specialized centers. We chose the term *medically supervised fitness training*, rather than CR for this level, as the term “rehabilitation” may be perceived by some as a treatment to aid in the recovery from an acute debilitating event (e.g., myocardial infarction or stroke); whereas many children and adolescents with CHD have chronic impairment in fitness and physical function that has been present throughout much of their lives.

The constructs described in the CET intervention approaches are not in-themselves novel in the CHD population. Assessment of PA and fitness, promotion of PA, exercise prescription, and supervised fitness training interventions have been described individually in numerous publications, clinical trials, and textbooks. However, the interaction and congruence of each of these different tools and treatments in the CHD population is rarely discussed.

The CET model provides a framework for promoting and implementing strategies to improve fitness in patients with CHD. The application of existing, new, and emerging eHealth, mHealth, and commercially available and consumer-facing technologies may enhance delivery and success of each stage within the CET framework. Multiple intersecting inputs need to be considered when choosing the most appropriate technology to be implemented in each CET stage. These considerations include a thorough understanding of the goals of each stage to identify the technologies with the greatest specificity, potential of the technology to increase effectiveness and access, and the technology's degree of cost and resources required. Technology itself more broadly may be defined as “the application of scientific knowledge for practical purposes” (22), however, technology pertaining to CET has a narrower scope and may be considered to be the tools, products, and devices that enhance the use, delivery, effectiveness, and research of PA and exercise therapeutics. Technologies can be utilized across all CET stages, assisting providers in assessing their patient's PA and fitness levels, promoting PA, designing, and monitoring exercise prescriptions, and delivering fitness training interventions.

The goals and role of technology as they apply to each CET stage are described in Table 1. Technologies can overlap between each CET stage. For example, PA monitoring built into existing ubiquitous technology like consumer-facing wearables and cellular phones can be used for both the assessment and delivery of an exercise prescription. MSFT may include device-based measures of PA and exercise such as accelerometers and heart rate monitoring devices for tracking and monitoring adherence to the program, progression, motivation, and assessing effectiveness of the intervention. Online delivery platforms for exercise prescription and supervised fitness training may provide participants with educational modules to facilitate the intervention or promote a sense of community through socialization with peers. Similarly, mHealth and telehealth technologies for asynchronous or synchronous exercise prescription and supervised fitness training intervention programs may enable patients who are physically distant to benefit from care.

**Table 1:** CET: objectives and purpose of technology.

CET	Objective(s)	Purpose of Technology
PA and fitness assessment	To: (1) determine patients’ level of PA participation and fitness to identify the ideal intensity of the intervention; (2) establish measurable metrics to identify change following intervention.	Measurement/tracking, frequency, adherence, reliability of measurement **Health Equity:** Health resource utilization
**Intervention Tiers**		
PA and exercise promotion	To promote, educate, and encourage patients and families to increase or maintain habitual PA participation as described in the PA guidelines or diagnosis specific recommendations	Education, motivation, social connectedness, state of behavior change **Health Equity:** Education, expanding health resources
Unsupervised exercise prescription	To develop an individualized exercise prescription utilizing the FITT principle, that is designed to be: (1) self-administered (without direct medical supervision); (2) developmentally appropriate; and (3) executed using the exercise resources available to the patient and family in their home or community.	Tracking/monitoring (frequency, intensity, time, type, volume, progression), education, interactive exercise guides/workouts, motivation **Health Equity:** Opportunity, individualized prescription based on resources available
Medically supervised fitness training	To deliver an individualized exercise training program that is administered under the direct supervision of a clinical exercise physiologist and medical provider.	Safety monitoring, real time physiologic monitoring, delivery platform **Health Equity:** Access for patients far from a hospital facility or without reliable transportation

**Figure 2:**
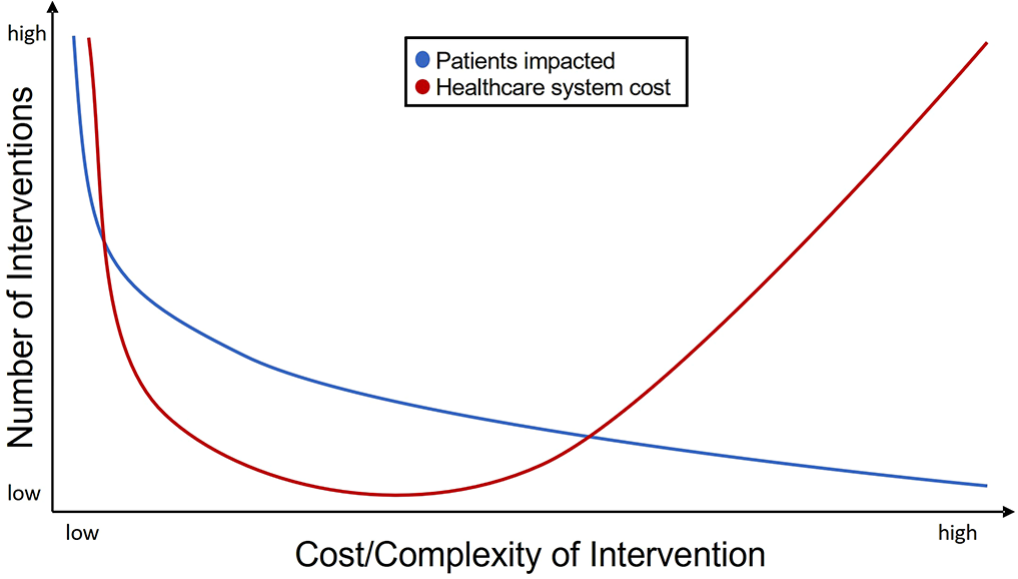
Theoretical schema representing CET intervention tiers, and resources, expertise, and relative numbers of patients to be served at each level.

Technology can be leveraged as a valuable tool to individualize treatment by tailoring the interventions based on patient hemodynamics, patient needs and learning styles, and family and environmental factors. Additionally, technologies can be evaluated and implemented at a health systems level, including the ability to scale for broad dissemination (i.e., PA promotion), as well as delivering precision CET for the patients needing the most intensive intervention. However, these applications are not without cost. Figure 2 represents a theoretical balance of cost between potential for dissemination and complexity/resource utilization for an individual patient and the chosen technology. Broad application of low cost and complexity interventions for many patients could result in high cost to the health care system given the potential for large number of patients that could be served and may raise questions regarding the overall value of these interventions. Inversely, only building capacity for low volume, high-complexity care may also pose a value-based challenge to health care systems. However, the middle of the cost-complexity relationship potentially represents a range that leverages the best utilization of technologies for the individual and for the health system by enabling amplification and remote access to expertise and opportunity, resulting in more equitable care in geographic regions where access may otherwise be limited.

## 3. Physical activity and fitness assessment

Over the past 5 years, both research and consumer-facing accelerometers and PA monitors have become more accessible and cost-effective eHealth technology options for PA and exercise surveillance. However, the utilization of wearable technology for assessing PA is still relatively unexplored in the CHD population, though the evidence base is expanding. Table 2 briefly describes the intended use, data tracked, and advantages and disadvantages of common wearable devices described in PA literature (23).

**Table 2:** Technology-based Assessments tools for CETs.

Device	Definition	Data tracked	Purpose	Advantages/Disadvantages
Heart rate monitor (watch, chest band, clothing, skin patch, smart rings)	A device with a sensor that detects and tracks heart or pulse rate continuously	Heart rate (beats per minute)	Provides live feedback on exercise intensity and duration	Relatively inexpensive, non-invasive and versatile, accuracy varies depending on device and location worn
Pedometer (commonly worn on wrist, belt or shoe)	A device with a motion sensor that detects movement	Steps (distance covered by foot)	Provides an objective measurement of PA level by showing distance traveled	Relatively inexpensive, easy to use, increases PA awareness, and can be used as a motivational tool. Accurate for walking but not for jogging or running, can be manipulated by shaking the device, which can overestimate PA. Cannot be used for swimming activities.
Research grade Accelerometer (ActiGraph most common in children and adolescents, device can be worn on a waist belt)	A device with a motion sensor (using piezoelectric transmitters) that detects acceleration of the body in different planes	Body movement in counts per unit of time (referred to as an epoch). Rate and intensity of movement is tracked	An objective measurement to quantify daily PA and sedentary behavior	Relatively small in size, large capacity to record data continuously over an extended period of time, greater accuracy and precision, ability to quantify time spent in different activity intensity levels. More expensive, more expertise needed to operate, lack of visual feedback to individual wearing the device, uniaxial devices can be less reliable
Consumer-facing PA monitors (wristbands, smartwatches, chest straps, clothing)	A consumer-facing wearable device developed for the general population that tracks and estimates energy expenditure across a wide range of activities	Step count (distance), caloric energy expenditure (calorie count), heart rate/rhythm, sleep quantity/quality	Provides objective data on energy expenditure and heart health	Objective measure of PA level, reminders to move, can help with motivation, community connection/support, consistent tracking for progress, set goals, tracks sleep and heart rate and easy to use. Not always a reliable/accurate measure of calorie expenditure and distance tracked, can be expensive depending on the device and most devices have limited battery life

Recall questionnaires and self-report PA surveys have been a somewhat effective tool for assessing PA in the CHD population over the past two decades. Research by Voss and colleagues studied the Physical Activity Questionnaire for Children (PAQ-C) in children and adolescents with CHD and found the PAQ-C summary score was significantly correlated with total PA (counts per day), PA intensity, moderate-to-vigorous intensity PA (MVPA), vigorous intensity PA, and sedentary time measured with waist-worn, research grade ActiGraph accelerometer (24). Although self-report PA questionnaires can provide some estimates of PA, device-based assessments of PA present an objective and more accurate estimate of PA participation when compared to self-reported PA. A review by Skovdahl and colleagues of 15 studies that examined various methodologies in the assessment of PA in patients with CHD, the authors found wide variations self-reported PA when compared to healthy peers, but no between-group differences when PA is measured with wearable devices (25). The authors suggest methodological variation and limitations in self-report assessment may be responsible for these differences.

Short, sporadic, and high-intensity bouts of PA are difficult to quantify via recall questionnaires, and youth tend to over-estimate PA when self-reported on valid and reliable questionnaires (4, 25). Device-based measures of PA allow clinicians and researchers to perform well-described analysis methods providing insights into specific patterns of PA, including weekly frequency, bout pattern, intensity metrics and daily timing (26–28). For example, White and colleagues compared PA patterns in children and adolescents with Fontan circulation, pulmonary hypertension, and heart failure to cardio-normal peers using waist-worn ActiGraph accelerometers over 7-days (28). The authors did not observe any between-group differences for sedentary time or total PA, however, they reported differences in light intensity and MVPA. Additionally, they reported participants with CHD accumulated MVPA in more sporadic (<5 min) bouts, where their peers without CHD accumulated a greater proportion of MVPA in short (5–9 min) and medium-long (≥10 min) bouts. Lastly, the authors noted that both groups engaged in similar levels of PA in the mornings and midday, but the CHD participants were much less active in the afternoons, particularly between 3–5pm (28). Detailed analysis of PA using research grade accelerometers may provide insights into PA promotion and intervention that are not possible with self-report.

Researchers and clinicians should be cognizant of the potential for methodological error when measuring PA with self-report or wearable devices. Skovdahl et al. noted that that the utilization of accelerometry to measure PA can be influenced by measurement protocol, device settings, body placement, processing of raw data, method of calibration and statistics (25). Lack of training of study personnel resulting in participants receiving inadequate instructions and anchoring to the device often results in limited device wear-time, reducing the quality of the data and ability to apply validated intensity cut-points.

Assessing PA using consumer grade PA monitors require less training, devices are less expensive, and data can be collected remotely. Institutions can purchase a specific application programming interface (API) from companies such as Fitbit or Garmin. The API allows for remote retrieval of data and events from the wearable. Data is presented similarly to the app-based interface, where it is aggregated into predefined metrics such as steps, heart rate, or activity minutes; though it should be noted that many of these metrics are determined using proprietary algorithms and researchers should interpret these results with caution. Using an API, surveillance of PA can be essentially “touch free,” though participants are still required to sync their device to an internet enabled device. A more advanced and more expensive method to monitor PA remotely is to purchase the software development kit (SDK). The SDK makes it possible to create custom programs to communicate with the device to retrieve data and manipulate the device remotely (29). Use of API or SDK technology requires technical expertise and the institution's information technology department should be consulted. Additional details on API and SDK interfaces with consumer-facing PA monitors can be found in Henriksen et al. (29). Implementation of these approches will require expertise from individuals who can understand coding and software development, working in conjuction with exercise physiologists or other experts in the measurement of PA. Institutions can also partner with companies such as Fitabase, which provide research grade interfaces with consumer-facing activity monitors through an API.

Consumer-facing PA monitors have several limitations. Voss and colleagues equipped 30 children and adolescents with CHD with the consumer-facing wrist-worn Fitbit Charge HR PA monitor and a research-grade waist-worn ActiGraph accelerometer (30). The authors found a correlation between the two devices for daily steps, though the consumer-facing device overestimated steps by an average of 2,242 per day (absolute percent error of 28%). Additionally, the consumer-facing device tended to under-estimated MVPA in some participants and over-estimated MVPA in others (30). Most consumer-grade PA monitors are designed for adults, who often participate in prolonged and continuous bouts of exercise (30). For example, Fitbit devices only records MVPA active minutes that are accrued in bouts of ≥10 min (30, 31). Evidence suggests that children and adolescents with CHD accumulate less than 1% of their MPVA in medium-long bouts of ≥10 min, and 91% in sporadic bouts of <5 min (28).

Other non-traditional devices such as cardiovascular implantable devices can provide estimates of PA for cardiac patients using embedded single-axis accelerometers (32, 33). A 2016 study by de la Uz et al. was one of the first to describe this approach in a sample of 1,905 children and adolescents with an implanted cardiac device. The authors extracted 4-weeks of PA data at 53-weeks post-implantation and aggregated PA data by counting the number of minutes above the activity threshold, which includes the number and magnitude of deflections in the accelerometer signal (34). After multivariate modeling, the de la Uz and colleagues observed male gender, device type (pacemaker), and device location (epicardial) had the largest effects on higher PA participation (34). A similar study by Przybylski and colleagues assessed movement patterns derived from pacemakers, implantable cardioverter defibrillations and implantable loop recorders to estimate PA in children prior to and during the COVID-19 pandemic. The authors acquired PA data from databases kept by the device manufacturers (Medtronic and Boston Scientific) or reports available through electronic medical record (EMR) and were able to observe a decrease in PA derived from accelerometers within the cardiovascular device associated with implementation of social-distancing policies in the patient's region (33).

A 2018 systematic review of studies measuring PA with implanted cardiac devices only identified one study using this modality in youth and no studies specific to congenital heart disease (34). Thus, this modality has not been formally utilized as a PA assessment tool to inform recommendations or exercise prescriptions in pediatric CHD populations, despite the data being readily available. No formal validation studies in youth and low device prevalence in pediatric populations relative to adult cardiac populations may be a barrier to implementing PA monitoring using implantable cardiac devices, but this may be another area for further growth. Using implanted cardiac devices for assessing PA is not without limitations. For example, the authors reported that some implanted devices were limited by collecting a maximum of 8 h of movement per day. Additionally, PA data collected from implantable devices is limited by the precise threshold of movement necessary for the device to recognize a bout of PA (proprietary information from the manufacturer). These data are binary in nature and do not discriminate between PA just below the pre-set activity threshold or highly intense PA (33).

Adequacy of PA levels may also be assessed by cross-sectional or longitudinal physical fitness assessment. Physical fitness is comprised of five measurable components: cardiorespiratory function, muscular strength, muscular endurance, flexibility, and body composition (35). Assessment of fitness can provide insights into PA, exercise prescriptions, and fitness training interventions. Cardiopulmonary exercise test (CPET) and assessment of PA, QoL, and anthropometrics (e.g., BMI) have been commonly reported in the CHD and exercise literature. Translation of novel PA and fitness assessment methods and technologies in the health-fitness, sport performance, obesity, and diabetes fields has provided the CHD field with a new set of tools that can be used to standardize clinical outcome metrics and assessments. The frailty paradigm, translated from research in geriatric populations, is an emerging low-tech assessment tool in the CHD population. Frailty is a multi-component assessment of physical function that includes measures of slowness, weakness, body composition, fatigue, and PA. Assessments of the five components are aggregated into a “frailty score” that has been associated with short and long-term outcomes in youth with CHD (36, 37).

Consumer-facing PA monitors, such as Apple watches, and some FitBit and Garmin devices combine tri-axial accelerometers, heart rate sensors, pulse oximeters, and barometric pressure to estimate an individual's functional capacity to evaluate physical fitness progression and potentially improve performance (38). In a systematic review by Passos et al, the authors aimed to identify and summarize literature that used wearables and “Internet of Things” (IoT) technologies for fitness assessments in athletes. There were eleven specific wearable/IoT devices that were examined and categorized into four relevant domains for implementation and impact: sensing, processing, communications, and applicability. Studies in the applicability domain reported results for fitness assessments and identified two important categories: physiological status monitoring and activity recognition/tracking. Physiological status monitoring includes variables such as sweat rate, heart rate, and when combined with activity recognition/tracking from an accelerometer, data have been used to assess exercise training and intensity zones. Further combinations of these variables could present new technological approaches to individualize and optimize exercise training. For example, hydration replacement at certain heart rate/intensity zones and assessing the combination of heart rate and acceleration data may aid coaches in better understanding an athlete's baseline fitness level and the dose-response relationship between training (dose) and fitness (response) (38). Some of the more novel approaches include using consumer-facing devices to measure continuous heart rate and PA monitoring, step count, and sleep patterns which were sent to a cloud platform where a human “Digital Twin” was created. Once an adequate amount of data is collected, the “digital twin” algorithm is able to predict the athlete's performance and identify when changes to the training program are needed (39). The authors note that specific sensor technology, such as inertial sensors and the use of a depth camera, as well as chest bands with accelerometers, can also be helpful in injury recognition and prevention by assessing athletic behaviors on and off the field and body posture when performing exercise (38). Although research using wearables for assessing PA and fitness in the CHD population is still in its infancy, these kinds of advanced methods for predicting PA and fitness may be clinically applicable in the future.

As new and emerging PA and fitness assessment and intervention technologies are translated to the CHD population, institutions will likely need to invest in resources. Additionally, many of these technologies will need to be both patient and provider facing and valid and reliable for this unique population.

## 4. Physical activity promotion

Despite the physiological and psychosocial benefits of regular PA and exercise, youth engagement in PA remains an issue. A study using data from the 2018 Youth Risk Behavior Surveillance System reported that less than half of children and only 8% of adolescents were achieving appropriate amounts of PA (8). Unfortunately, adherence to PA and exercise recommendations are lower in children and adolescents with CHD compared to their cardio-normal peers (9, 10, 28). Promotion of PA is a challenge faced by all pediatric providers. However, considering reluctance and fear of injury related to PA in patients and caregivers of youth with CHD, PA promotion in this population is perhaps even more complex (1, 3–6).

Effective and evidence-based strategies that target promotion and support change in habitual PA in children and adolescents require advocacy and leadership by administrators, medical providers, and/or exercise physiologists (11, 19). Well-defined strategic recommendations to enhance PA participation among the CHD populations include several key components, such as: (1) assessing readiness and the ability to change; (2) understanding the specific types of activities that should be encouraged; (3) assessing what clinical considerations are relevant to an individual's PA; (4) clear messaging about recommendation; (5) personalized exercise prescriptions, and (6) regular follow up to ensure adherence (1, 4, 20).

Communication and mHealth technologies, motivational applications, remote monitoring, and wearable tracking devices have a high potential for dissemination and may have a large impact on PA promotion (22). Consumer-facing PA monitoring technology including computer and mobile applications (apps) and wearable devices from companies like Apple®, Garmin®, Fitbit®, Polar® etc. provide patients with direct and instantaneous feedback, are relatively inexpensive, and can be monitored remotely using company specific APIs. eHealth and mHealth technologies may provide several benefits over traditional PA promotion techniques (e.g., educational materials, in-office counseling) including improved adherence, greater ability to remotely access PA data, counselling, providing real-time feedback, and youth's preference for using technology for school, social, recreational, and other tasks (40, 41).

Promoting PA utilizing wearable technologies, without additional behavior change strategies, may have limited effects. A 2016 literature review by Ridgers et al. found a paucity of evidence endorsing wearable tracking devices and other technology as tools for promoting PA in children (42). The authors observed no significant lasting differences in PA in youth using wearable activity trackers when compared to PA without wearable activity trackers (42). However, emerging research suggests a positive influence of technology on promoting pediatric PA using modalities such as gamification (40, 43–45). The term “gamification” refers to the use of game design elements in non-gaming contexts to engage participants, encourage health learning behavior, and practice positive health behaviors to create a more creative and playful engagement which overall has a positive and motivational influence (46, 47). In addition to wearables, a 2019 review of the efficacy of PA promotion in adolescents via mHealth technologies (specifically websites, wearables, apps, and combinations of these three) demonstrated these strategies were effective at improving PA outcomes over time especially when paired with in-person sessions (48). However, the authors noted weakness in the literature and called for better study designs, better defined outcomes, and the involvement of adolescent participants when designing the study and determining how the technologies will be used (48). For example, a 2017 study by Mendoza et al. using Fitbit® and Facebook to promote PA in adolescent and young adult childhood cancer survivors did not demonstrate an increase in MVPA (49). Participants in this study provided feedback, suggesting Facebook was not routinely used by the targeted age group, and participant engagement in the development of the intervention may have improved adherence (49).

One hallmark of adolescence is the increasing salience of social and community-based behavioral reinforcement and emotional rewards. For patients with CHD, social inclusion may be impacted by limited or restricted PA and may be a contributing factor to the increased risk for depression, anxiety, and lower resilience found in pediatric heart disease populations (50–53). Children as young as 5 years old have shown modulation of PA to match their peer's patterns (54). The LOSE-IT trial demonstrated that sharing weight loss progress with a self-selected teammate was just as effective as interventions that included more complex, gamified behavioral modification strategies (55).

Psychosocial factors have a major influence on inactivity, perhaps more than the severity of the illness or chronic condition. Structured approaches to address psychosocial barriers to PA participation is vital for adoption of new PA habits (56). eHealth technologies can have a role in facilitating behavioral change and subsequently PA promotion (57, 58) and have already been associated with improvements in the promotion of PA and adherence to PA programming in adults (59, 60). However, the data supporting technology as a tool for PA related behavior change in children and adolescents is still emerging. A 2014 systematic review of PA apps by Brannon et al. assessed child and adolescent behavior change techniques and found that a health coach modeling a health behavior was the only positive predictor of PA behavior change in children (6–13 years of age) (61). For adolescents (≥13 years of age), increased PA behavior was influenced by consequences (providing information focusing on what will happen if the person performs the target behavior, including the benefits and costs of action or inaction), behavioral contracting, other's approval, self-monitoring, and intention formation (61). Of the apps studied, 62% included some component of health behavior modeling, primarily via app incorporated videos, 42% incorporated self-monitoring, and 1% incorporated intention formation; however, none of the apps studied incorporated behavioral consequences, contracting or information on external approval (61). Based on these limited findings, the authors concluded that apps were not as beneficial for promoting PA in adolescents relative to children (61).

Technology, with its wide range of potential benefits, is an enticing option for PA promotion; however, there is a paucity of research that specifically addresses the use of various technologies for PA promotion in children and adolescents with CHD. Budding data within the CHD population supports implementation of fitness-tracking technology to assess adherence to physical activity interventions (44). Jacobsen et al. used the consumer-facing Fitbit Flex® activity tracker to assess adherence to a home-based exercise program for Fontan patients aged 8–12 years old (44). In this intervention, participants could join a secure online group through the Fitbit® website that allowed participants to compare their progress to other study participants (44). This peer support element incorporates behavior change techniques that have been effective in other adolescent PA promotion studies (61).

Promotion of PA remains a challenge among the pediatric population including those with CHD. Awareness of available technology for pediatric PA promotion can be extremely valuable, but the clinician/research should be aware of the weaknesses of these methods, should directly involve patients (specifically including children and adolescents) when identifying technologies, and include technologies that utilize developmentally appropriate behavior change techniques. Additional studies are needed to evaluate the use and effectiveness of technology for PA promotion in patients with CHD. The integration of established technology, as outlined, has the potential to enhance PA promotion efforts, personalize exercise routines, and educational materials provided within a cardiac rehab program. For maximum effectiveness, it is imperative to select technologies that have been thoroughly evaluated and understood by medical providers and exercise physiologists while also considering the patient's age and technological proficiency when selecting appropriate technologies and behavioral motivation techniques. With proper utilization, established technologies can complement physical activity promotion efforts and lead to improved patient outcomes.

## 5. Unsupervised exercise prescription

An exercise prescription for patients with CHD is an individualized plan to participate in exercise, guided by diagnosis specific recommendations for PA and patient/family factors. Like medication-based therapies, the patient is prescribed a specific “dosage” of exercise, where the dosage is described using the FITT principal framework. The FITT principle describes an exercise prescription under the categories of Frequency (days per week the exercises will be performed); Intensity (light, moderate, or vigorous); Time (duration of the exercises), and Type (exercise modality) (62). An exercise prescription should be informed by the medical evaluation and resultant risk classification. For patients with cardiac disease this includes, but is not limited to, ventricular function, presence/degree of hypoxemia, rhythm abnormalities, coronary risk, pressure and volume load, and other considerations related to complex physiology (63). The exercise prescription can be tailored to limit or promote specific types and intensities for patients with medically necessary exercise restrictions. However, daily exercise is recommended and considered safe for most with CHD.

Written and detailed exercise prescriptions designed by trained exercise physiologists or other trained personnel may be more effective and have greater adherence than verbal recommendations for increasing PA (20). Well-designed, unsupervised, in-home exercise prescription have been shown to be effective in improve fitness and QoL in patients with CHD (44, 64). For example, Morrison and colleagues provided an exercise-based motivational session followed by a 6-month self-implemented exercise prescription in a sample of adolescents with CHD and concluded that unsupervised exercise training can produce significant improvements in fitness levels and daily PA (65).

The utilization of eHealth and mHealth based education interventions for exercise prescription mirror those described in the PA promotion section. The effect of education alone or as part of a multi-faceted approach to behavior change is complex, differing across age groups as best studied in the pediatric obesity literature (66–68). While educational interventions alone are unlikely to provide a satisfactory impact on cardiovascular outcomes, they are an important component of all exercise prescriptions and interventions. Well powered randomized control trials evaluating the safety, quality, and efficacy of specific educational platforms for patients with CHD have not been published to date. Most online educational resources specific to cardiac patient populations are created for adults with content specific to disease states that are less common in pediatric populations. Optimized and evidence based educational exercise programs may require consultation child psychology or others with expertise in education. Research in the development of program content and structure optimized for a pediatric cohort is still in its infancy.

Many consumer-facing smartphones, smartwatches, and other wearable technologies have available APIs or can link to internet applications, allowing the care team to remotely assess adherence to the frequency and duration components of the exercise prescription. The care team should be cognizant of the limitations of these devices and design the exercise prescription accordingly. For example, some evidence suggests that consumer-facing wearable devices may have difficulty identifying an exercise bout if the patient fails to sustain a steady state (at least 5 continuous minutes of movement) (69). Additionally, accuracy of wrist-worn devices to capture a bout of exercise is compromised when participating in activities with less wrist motion such as cycling (70). Similar inaccuracies in wrist-worn activity monitors have been observed with resistance training (71). The patient selecting the “exercise mode” may aid in identifying that an exercise session was initiated at a certain day and time. Additionally, selecting the “exercise mode” on the wearable device may increase the accuracy of these devices to capture duration and intensity (heart rate) of the exercise session (70). The ability to monitor heart rate enables the patient to self-monitor their exercise session and stay within the prescribed intensity range and allows the care team to tailor the exercise prescription to the patient's relative functional capacity. The accuracy of consumer-facing devices for measuring heart rate in children and adolescents, regardless of CHD, has not been adequately studied.

Consumer-facing fitness technologies may aid in increasing adherence to in-home exercise prescription (72, 73). Many devices include automated feedback and gamification methods like badges, streaks, levels, and social engagement mechanisms to maintain enthusiasm and compliance. In anecdotal experiences, some individuals attribute new or increased positive health behaviors to the use of the device. No randomized studies exist on the impact of wearable devices for exercise prescription in the pediatric CHD population. However, a large and well-designed 24-month randomized-controlled trial in 471 young adults demonstrated less weight loss in the group assigned to a wearable activity tracker intervention compared to traditional behavioral change methodology (74). There is growing potential of wearable devices for delivering exercise prescription, but understanding optimal device implementation, specific gamification mechanic selection, and potential for unforeseen consequences in the clinical setting requires further study (47).

An increasing number of exercise-focused mobile applications for smartphone and smartwatches provide seemingly endless options for exercise type or modality. Many of these apps are free or low-cost and provide easy access to exercise-based streaming services, allowing patients and the care team to work together to identify exercises that align with their goals. Many apps provide a mechanism for tracking quantity of activity, which can provide feedback to both patient and provider and can inform progression of the prescription as the patient's fitness improves. However, most of these apps are focused on adults without chronic disease and at this time there are not many user-friendly applications for pediatric patients that are accessible without parent assistance.

eHealth technologies may improve adherence to exercise prescriptions as they may incorporate behavioral change strategies to structured interventions in adolescent and young adult populations (75). Effective application of eHealth technologies developed by a multi-disciplinary group including exercise physiologists and psychologists may improve self-monitoring skills and can provide immediate feedback and insight into an individual's health and behavior habits. Studies in adults with chronic disease found increased rates of adherence to interventions with eHealth components but did not demonstrate consistent improvement in physiologic or anthropometric outcomes (76, 77). Reinforcement schedules, goal setting and review, and automated assessment of a patient's progress towards and progression through a predefined exercise prescription are all possible with various eHealth technologies.

Like PA promotion, gamification has been used in conjunction with modern technologies for clinical and commercial use to improvement adherence to exercise. Systematic reviews of gamification in eHealth applications report limited empirical evidence and large knowledge gaps, though low and moderate quality data focused on exercise have been reported (47, 78). Few studies have tried to separate the impact of transparent activity data sharing from the gamified mechanisms that eHealth sensors enable. The Leveraging Our Social Experiences and Incentives Trial (LOSE IT) was a 36-week randomized-controlled trial in young adults that compared a standard treatment group of weight loss teams, with smart scales that automatically shared weight loss with their self-selected teammate to a gamification group that also included points and levels. All study arms lost significant weight without any significant difference in the gamified cohorts (55).

Safety is significant consideration when patients with CHD participate in unsupervised exercise. Exercise prescription executed in the home and without direct supervision of a medical professional may be perceived by some patients with CHD and their families to be dangerous, possibly impacting adherence. Smartwatches and small portable telemetry technology may facilitate remote monitoring and provide a sense of safety when patients are exercising outside of clinical settings. Heart rate monitoring can be used to guide the exercise prescription and reduce patient and family anxiety by providing a metric that demonstrates they are exercising effectively but also that they are not exceeding a “safe zone.” As patients become acclimated to their exercise routine and learn to perceive their body's signals as “normal” sensations related to exercise, this remote monitoring technology may provide in-the-moment reassurance. Developments in eHealth technologies providing remote hemodynamic and rhythm evaluations may increase the utilization of exercise prescriptions across a wider range of patient ages, diagnoses, and risk profiles.

## 6. Medically supervised fitness training (MSFT)

A MSFT intervention is a structured exercise program, beyond exercise prescription, that is designed to provide advanced monitoring and support including some (or multiple) components of education, coaching, tracking, and social support. MSFT programs can be divided into three generalized care settings: outpatient facility, in-hospital, and in-home. To understand how eHealth technologic innovations may alter the effectiveness and outcomes of these programs, we must first understand how traditional CR programs have advanced into their current form.

The first American Heart Association statement on CR was published in September 1994 (79), but reports of improved cardiovascular function with MSFT in adult patients with ischemic coronary disease (80), after myocardial revascularization (81), and status post heart transplant (82) had been described for several decades. The modern adult CR program is designed to intervene directly upon the underlying causes of atherosclerotic cardiovascular disease in adult and geriatric populations, including: sedentary lifestyle, obesity, smoking, hypertension, insulin resistance, and hyperlipidemia (83). Development of comprehensive programs that include exercise training, nutrition counseling, smoking cessation, and medication management have transformed traditional adult CR into conduits for secondary prevention with well-defined quality and outcome measures (84). Highly effective adult CR programs consistently demonstrate mortality and morbidity reduction even when faced with enrollment, adherence, and sociodemographic barriers (85).

Traditional CR programs are typically divided into three phases. Phase I, in-hospital CR focusing on transitioning patients from an acutely unstable state towards home going progression and handoff to outpatient (Phase II) CR programs. Advancements in care have rapidly decreased lengths of in-hospital stay for most populations following an acute cardiac event, thus, the majority of CR is delivered in Phase II outpatient programs. The optimal Phase II CR program, as recommended by the CDC, consists of 36 one-hour sessions, including team-based, exercise training with physiologic monitoring and direct supervision, education and skills development for heart-healthy living, and counseling on stress and other psychosocial factors (86). Phase III CR consists of medically supervised exercise without physiologic monitoring and frequently includes home exercise instruction components. The CR framework utilized in adult populations has become the prototype for many MSFT programs in pediatric CHD.

In pediatric heart disease, the in-hospital setting (Phase I) is the most resource and technology intensive setting for delivering MSFT. Most facilities utilize physiologic monitoring and daily, in-person, supervised exercise or therapy/movement sessions with physical therapists, occupational therapists, or exercise physiologists. Technological advancements have resulted in a small but increasing population of children and adolescents supported by extracorporeal or implanted mechanical support devices such as ventricular assist devices (VADs) (87). Data in adult patients with VADs have demonstrated that participation in a CR is associated with a reduction in morbidity and mortality (88). Specific technology for the delivery of Phase I interventions, beyond intensive standard-of-care monitoring, has not been a primary focus in existing literature. Phase I MSFT interventions ranging from a focus on early mobilization to full exercise training programs for pediatric patients with VADs have rapidly advanced, in part, due to the identification of knowledge gaps and variation in care driven by inter-institutional knowledge sharing and outcome reporting (89, 90). Technology-informed, and technology-driven frameworks for standardization and implementation of Phase I MSFT interventions via multi-institution quality improvement efforts will be key components in studying the safety and efficacy of in-hospital interventions for this growing segment of the CHD population (91).

Like adults with cardiovascular disease, improvements in the surgical and medical management of CHD have resulted in decreasing lengths of in-hospital stays for most patients (92). As such, pediatric MSFT interventions typically occur in outpatient settings (Phase II). The transition from Phase I to Phase II MSFT programs often utilize an “assess and refer” structure. Research by Grace and colleagues suggest that greater utilization of outpatient adult CR programs for patients with short inpatient stays may be facilitated by improvements and modifications to EMR technology to include automated referrals and reminders for clinicians to refer patients (93, 94).

Outpatient (Phase II) MSFT interventions for CHD populations have a wide breadth of scope and structure. Due to the diversity in program structures between institutions, the application of technology in this space are wide ranging, including supervision and delivery, education, and guidance, monitoring and tracking for feedback and safety, and facilitating social connection. Although some Phase II programs were already exploring hybrid and virtual models, the COVID-19 pandemic forced a transition away from in-person MSFT and accelerated the development of exercise specific eHealth and remote monitoring technologies. Virtual MSFT programs may provide consumer-facing heart rate monitors and educational and demonstrative exercise videos to patients, while connecting them with their providers via online communication platforms to allow for home-based exercise training with supervision, tracking and coaching (44, 95–97).

The most reported real-time MSFT program approach includes live video streams of each patient while exercising in either a one-on-one or group formats (98). MSFT interventions combining telehealth and real-time monitoring technologies allow exercise sessions to have an element of medical or professional supervision while also making the intervention accessible to a broader scope of patients. An example of this approach is the Fontan Fitness Intervention Trial (F-FIT) proposed by Tran and colleagues (99). This trial is designed to compare traditional in-hospital MSFT, live telehealth MSFT (via Zoom®), and in-person MSFT delivered at a community sport center or fitness facility. Participants randomized to the telehealth group participate in three partially supervised group sessions per week and are provided with resistance exercise equipment and a heart rate monitor that is linked to a mobile application for intensity monitoring and coaching (99). A similar trial by Chen et al. used live videoconferencing software to deliver exercise training 3 days/week and nutritional counseling one day/week over a 12–16-weeks intervention period to 8–19 year old's with heart failure (100, 101). This telehealth MSFT resulted in excellent adherence and significant improvements in cardiorespiratory fitness and other cardiac and vascular health indices (100). Real-time cardiac telerehabilitation combined with additional digital health tools can expand on the effectiveness of a home-based program. For example, Maddison and his research team developed a smartphone application with features including heart rate data transmission, exercise performance review, goal setting, behavior change education, social support, and real-time communication with exercise specialists (102). The results demonstrated non-inferiority of the telerehabilitation platform compared to in-hospital fitness training; though the authors did not describe subjective or objective measures of patient acceptance, reliability, and utility of the individual technology components (102).

Remote patient monitoring has a strong track record of reducing morbidity and mortality in patients living at home with heart failure (103). These programs range from a multi-specialty team making telephone calls to connected scales and blood pressure cuffs for automatically importing home data into the EMR. Development of wearable sensors, both medical devices and consumer-facing, has broadly expanded clinical and research interest in real-time physiologic monitoring for MSFT interventions (104). Given rates of serious cardiovascular events in CR are low in patients with pediatric heart disease (105, 106), data supporting the effectiveness of these devices for identifying a serious adverse event during exercise training is limited.

Consumer-facing heart rate monitors and activity trackers do not feature real-time transmission of vital sign parameters to a clinical monitoring team but may be utilized by the patient during a video-connected exercise session. However, real time transmission of vital sign parameters during a virtual fitness session has been developed in at least one device, the MedBIKE. The MedBIKE is a video game linked ergometer designed specifically for pediatric and young adult patients with CHD. The MedBIKE platform allows for live medical supervision using a two-way audiovisual feed accompanied by telemetry, pulse oximetry, and symptom monitoring. Medical staff can modify the workload in real-time, allowing for either safe intensity progression or limitation, depending on the patient's performance. The MedBIKE pilot study safely engaged complex CHD patients in high intensity interval training (107). More research is required to understand the most efficacious use of real-time vital sign monitoring during exercise in the CHD population.

Participating in a MSFT program can be challenging, especially for patients who have not previously engaged in any kind of exercise program. mHealth and video-conferencing technology offers multiple opportunities for social engagement and virtual community building to support patients enrolled in a MSFT program. Early findings in the effectiveness of social media to support patients participating in a MSFT are mixed, but some studies have demonstrated positive effects (108). Social media can provide opportunities for peer support, educational information, accessible communication to medical staff, as well as encourage positive behavior tracking and self-assessment. Positive peer influence via social media can include support, relationship building, problem solving, behavior reinforcement, information sharing, and group communications through messaging platforms. Social game mechanics like leaderboards, competitions, and group activities may improve engagement and satisfaction scores but also risk negative rewards of failure to surpass others or not finding in-game friends to complete tasks (47). Weitzman et al. investigated the role of online social networks related to clinical or medical topics. They noted four key considerations regarding the quality and safety of online networks: (1) making sure that patients are receiving medical or health related information that is consistent with their care team's plan; (2) moderation or auditing of content for accuracy, transparency, and any conflicts of interest; (3) clear and readable privacy policies and the protection of information; and (4) patients having control over the sharing or publishing of their personal data (109). The potential for positive social reinforcement for patients enrolled in a MSFT program must be weighed against the risk of negative social experiences and well described associations between social media use and depression, anxiety, and body image issues in adolescents (110–112).

Due to the broad and widely varying nature of eHealth-informed MSFT, application of these technologies at an individual center or for a specific patient population must be approached with understanding of the specific goals and aims of the program, social and demographic factors impacting the target population and existing resources and expertise available at interested centers. Implementation of any eHealth MSFT will require expertise in managing PHI across multiple systems and understanding of the local, regional, and national data, safety, and age-based digital platform laws and policies.

## 7. Considerations for equity and accessibility

One of the most promising aspects of eHealth technologies is the possibility of expanding access, support, and inclusion of traditional SFT to underserved populations. Ongoing digital CR trials in the adult population have demonstrated improved enrollment equity using a home-based exercise program (113). Additionally, eHealth plays a major role in expanding timely access. Long wait lists for CR has been noted to be a major limitation of these kinds of therapies (114). Research in adult medicine demonstrated that even if every patient referred to CR attended all their sessions, CR centers in the United States could only accommodate 37% of the eligible population (115). mHealth and telerehabilitation approaches have been proposed as a possible solution for improving timely access to cardiac rehabilitation (116).

eHealth can also play a role in expanding access to subspecialty care. While access to subspecialty care has improved over the past decade (117) the sustained success of the CET model relies on access to these highly subspecialized teams. The few programs with a program dedicated to pediatric exercise interventions for patients with CHD are typically in urban settings. Video conferencing combined with wearable technology offers the potential to bring these highly specialized programs to those living at a distance from a program. The importance of easy access has been acknowledged and multiple programs have an in-home mHealth component, which has improved patient access to subspecialties throughout pediatrics (118, 119).

Major barriers to access MSFT programs are cost, caregiver burden, and travel time. Studies in adult populations quantified that patients participating in cardiac telerehabilitation may save $1,150 in travel costs compared to in-person CR (120, 121). A longitudinal study found 2.8 million U.S. dollars were saved in travel costs through the use of telehealth (121, 122). The use of mHealth technologies decreased the number of unexpected hospital readmissions and days in the hospital in children with a chronic medical condition by 50% which resulted in a cost reduction of $9,425 per patient in a group of children with chronic medical conditions (123). Reduced stress levels for caregivers of children with chronic medical conditions with the availability of programs via telehealth has also been described (124). mHealth technology options reduce cost and travel time and increase access to subspecialty care, leading to a decreased burden on caregivers.

Telerehabilitation has also been expanding globally. Several groups have studied the use of telerehabilitation in rural communities across the globe and found increases in compliance and access (125–128). However, in the work by Buyting et al., people in urban communities used telerehabilitation more than those in rural communities, signifying that there is more than just cost, time and transportation that limits people's use of telemedicine (125).

Limitations to using mHealth technology to expand access to those in rural areas populated by underrepresented groups and economically disadvantaged include poor or no internet access, lack of equipment and the expertise and financial costs required to develop programs which can be accessed from home and tailored to meet the needs of a diverse population. According to Pew Research, 96% of people 18–29 reported that they own a smart phone, only 76% of people with an income of <$30,000 reported owning a smartphone (129). This discrepancy demonstrates a large gap in access to the technology often used by telerehabilitation programs. To combat this access barrier, programs would need to incorporate the cost of tablets for those without a smart phone, Wi-Fi internet sticks for those without access to Wi-Fi or hotspots, equipment (such as a folding stationary bike to accommodate people in apartments), and a portable ECG device. Programs would have to negotiate this overhead cost into the insurance/Medicaid billing reimbursement to make telerehabilitation accessible to those in lower socioeconomic groups.

Beyond program costs, there are particular barriers related to cross-state licensing for health care providers in the United States. Legislation needs to allow patients in states without programs to access programs in other states without the prohibitive cost and time of travel. The COVID pandemic advanced the utilization of telehealth programs and many practitioners, especially at large urban institutions, are now comfortable with the current technology and have-hospital based telehealth programs in place to support utilization of this technology. Beyond financial burden there are also the security and privacy considerations related to eHealth and mHealth technologies. There are limited eHealth tools that allow for the transfer of exercise data to a secure and available clinical EMR platform. HIPPA compliant high-value eHealth platforms that allow for the transfer of wearable data to the EMR or health dashboard is essential for the expansion of telerehabilitation in the pediatric populations (75). A cost effective collaboration with experts in the technical space to develop and maintain secure a system would provide the resource needed for a secure platform, due to the complexity and constantly changing nature of the digital and online threats (130). These limitations are not small obstacles and need to be addressed in order for technology to truly help expand the reach of health care providers to the pediatric cardiac rehabilitation population.

## 8. Summary of future directions and conclusions

Looking to the future, there are multiple opportunities for today's and tomorrow's technology to assist in the support and enhancement of CET in children and adolescents with CHD. Long-term success of these programs will require an investment in PA promotion programs, increased utilization of individualized exercise prescriptions, and development of MSFT programs nationwide to fully commit to increasing physical activity and fitness in our patients. Currently there are a limited number of institutions offering CET intervention approaches. To meet the needs of the patient population, there needs to be growth of these programs and development of others. Additionally, improved education regarding exercise interventions in CHD is needed. A robust exercise education is almost non-existent in most pediatric residency and pediatric cardiology fellowship programs currently. There is a need to incorporate education of PA promotion, monitoring, prescription, and intervention into the training of pediatric providers to improve the care of our patients. As part of that education, physicians and other healthcare professionals must understand the technologies that are available to patients and how that technology can better connect them and provide data about their patient's fitness health. eHealth and mHealth technologies have provided for a platform to share and connect providers with their patients. This is of great importance as the population of those with CHD is small and dispersed. Further study is required to understand the need for continuous monitoring during exercise in our population. Privacy concerns related to social networking, collection of location data, journals, and symptom logs must always be considered. The development of these data sets for clinical use demands data protection and privacy controls as a foundational element is to maintain patient and institutional trust (109). Collaboration with experts in developing and maintaining secure systems is recommended due to the complexity and constantly changing nature of the digital and online threats (130).

The implantation of the CET components in a pediatric cardiology program will depend on the resources, expertise, and personnel available. However, implementing technology to CET may not require the institution to have an existing/formal cardiac rehabilitation program in place. Multi-disciplinary partnerships between physicians, nurse practitioners, exercise physiologists, physical therapists, psychologists, and information technology specialists may be necessary depending on the institution's approach.

Realistic objectives combined with a bold and expansive long-term vision utilizing national and international collaboratives and with adequate government support are most likely to push the use of eHealth and mHealth technologies into the future. A shift in healthcare models towards optimization of lifelong comprehensive health that includes PA and fitness should be standard component of CHD treatment. Utilizing technology to help monitor and deliver this care may not only improve effectiveness of these treatments, but also improving equity and access to patients in low-resource settings and underserved communities.
